# In Vitro and In Silico Study of Analogs of Plant Product Plastoquinone to Be Effective in Colorectal Cancer Treatment

**DOI:** 10.3390/molecules27030693

**Published:** 2022-01-21

**Authors:** Halilibrahim Ciftci, Belgin Sever, Firdevs Ocak, Nilüfer Bayrak, Mahmut Yıldız, Hatice Yıldırım, Hasan DeMirci, Hiroshi Tateishi, Masami Otsuka, Mikako Fujita, Amaç Fatih TuYuN

**Affiliations:** 1 Department of Drug Discovery, Science Farm Ltd., Kumamoto 862-0976, Japan; hiciftci@kumamoto-u.ac.jp (H.C.); motsuka@gpo.kumamoto-u.ac.jp (M.O.); 2 Medicinal and Biological Chemistry Science Farm Joint Research Laboratory, Faculty of Life Sciences, Kumamoto University, Kumamoto 862-0973, Japan; belginsever@anadolu.edu.tr (B.S.); htateishi@kumamoto-u.ac.jp (H.T.); 3 Department of Molecular Biology and Genetics, Koc University, Istanbul 34450, Turkey; hdemirci@ku.edu.tr; 4 Department of Pharmaceutical Chemistry, Faculty of Pharmacy, Anadolu University, Eskisehir 26470, Turkey; 5 Faculty of Medicine, Kocaeli University, Kocaeli 41001, Turkey; 200601195@kocaeli.edu.tr; 6 Department of Chemistry, Faculty of Engineering, Istanbul University-Cerrahpasa, Istanbul 34320, Turkey; nbayrak@istanbul.edu.tr (N.B.); hyildirim@istanbul.edu.tr (H.Y.); 7 Chemistry Department, Gebze Technical University, Kocaeli 41400, Turkey; yildizm@gtu.edu.tr; 8 Department of Chemistry, Faculty of Science, Istanbul University, Istanbul 34126, Turkey

**Keywords:** plastoquinones, colorectal cancer, cytotoxicity, apoptosis, DNA cleavage, molecular docking, pharmacokinetic properties

## Abstract

Plants have paved the way for the attainment of molecules with a wide-range of biological activities. However, plant products occasionally show low biological activities and/or poor pharmacokinetic properties. In that case, development of their derivatives as drugs from the plant world has been actively performed. As plant products, plastoquinones (**PQs**) have been of high importance in anticancer drug design and discovery; we have previously evaluated and reported the potential cytotoxic effects of a series of **PQ** analogs. Among these analogs, **PQ2**, **PQ3** and **PQ10** were selected for National Cancer Institute (NCI) for in vitro screening of anticancer activity against a wide range of cancer cell lines. The apparent superior anticancer potency of **PQ2** on the HCT-116 colorectal cancer cell line than that of **PQ3** and **PQ10** compared to other tested cell lines has encouraged us to perform further mechanistic studies to enlighten the mode of anti-colorectal cancer action of **PQ2**. For this purpose, its apoptotic effects on the HCT-116 cell line, DNA binding capacity and several crucial pharmacokinetic properties were investigated. Initially, MTT assay was conducted for **PQ2** at different concentrations against HCT-116 cells. Results indicated that **PQ2** exhibited significant cytotoxicity in HCT-116 cells with an IC_50_ value of 4.97 ± 1.93 μM compared to cisplatin (IC_50_ = 26.65 ± 7.85 μM). Moreover, apoptotic effects of **PQ2** on HCT-116 cells were investigated by the annexin V/ethidium homodimer III staining method and **PQ2** significantly induced apoptosis in HCT-116 cells compared to cisplatin. Based on the potent DNA cleavage capacity of **PQ2**, molecular docking studies were conducted in the minor groove of the double helix of DNA and **PQ2** presented a key hydrogen bonding through its methoxy moiety. Overall, both in vitro and in silico studies indicated that effective, orally bioavailable drug-like **PQ2** attracted attention for colorectal cancer treatment. The most important point to emerge from this study is that appropriate derivatization of a plant product leads to unique biologically active compounds.

## 1. Introduction

Chemotherapy is the main cancer treatment, whereas the drugs used in this treatment are no longer effective because of the drug resistance in cancer cell lines and /or undesirable side effects due to the lack of selectivity for cancer vs. normal cell lines [1]. For this reason, in order to cope with this disease, the development of new drug candidates with new therapeutic strategies based on natural products is urgently needed [2,3]. Many of the second metabolites are chemotherapeutically useful natural products which are the thriving source of anticancer drug discovery. For example, many compounds from nature demonstrate potent structures and remarkable activities, in particular anticancer properties [4,5,6]. Structure-based design from bioactive natural products is one of the most promising and fundamental approaches to generate novel selective biologically active molecules with favorable pharmacological properties [7,8]. For clinical use, most approved small anticancer molecules (around 83%), 113 of the total 136 molecules, are generated from natural products or inspired from natural products [9]. The importance of natural products would be that not only they can act on maximum efficacy in most cases and quench adverse effects, but they also are capable of multi-targeting [10].

**Figure 1:**
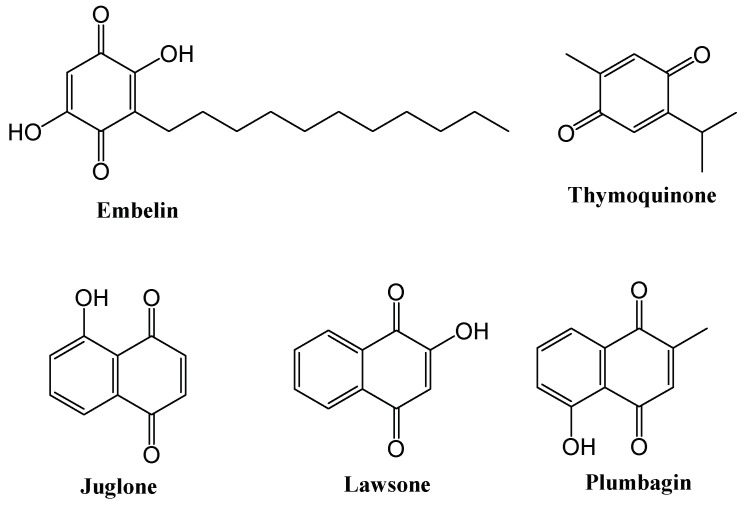
Quinone-based plant products with anti-colorectal cancer properties.

Over the last years or so, the use of 1,4-quinone, an important source of natural products, has enabled the design of efficient and active molecules with improved pharmacological properties in the search of new molecules with cytotoxic activity [11,12,13,14,15,16]. The 1,4-quinone and 1,4-naphthoquinone moieties are well-represented in bioactive natural structures and their anticancer effects, including anti-colorectal cancer properties, have been documented in several studies. Among these compounds, embelin (2,5-dihydroxy-3-undecyl-1,4-benzoquinone) (Figure 1) is isolated from the fruit of the herbal plant *Embelia ribes* and its antitumor effects have long been known. Embelin was shown to lead a significant decrease in tumor growth in mice, which were treated with the mixture of embelin and murine B16-F1 melanoma cells or LS174T human colon carcinoma cells [17]. Thymoquinone (2-isopropyl-5-methyl-1,4-benzoquinone) (Figure 1), one of the most important molecules isolated from natural product *Nigella Sativa* L., displayed an antiproliferative activity towards a range of cancer cell lines, including colorectal cancer cells [18,19]. Juglone (5-hydroxy-1,4-napthoquinone) (Figure 1), a natural component isolated from walnut trees, has been reported to inhibit tumor proliferation and metastasis and induce apoptosis in different types of cancer such as colorectal carcinoma [20,21,22]. Lawsone (2-hydroxy-1,4-naphthoquinone) (Figure 1) and plumbagin (5-hydroxy-2-methyl-1,4-napthoquinone) (Figure 1) are other 1,4-naphthoquinone-based natural products with potent anti-colorectal cancer effects [23]. Further to that, the structure–activity relationship elucidation paves the way to the foundation for lead optimization.

**Figure 2:**
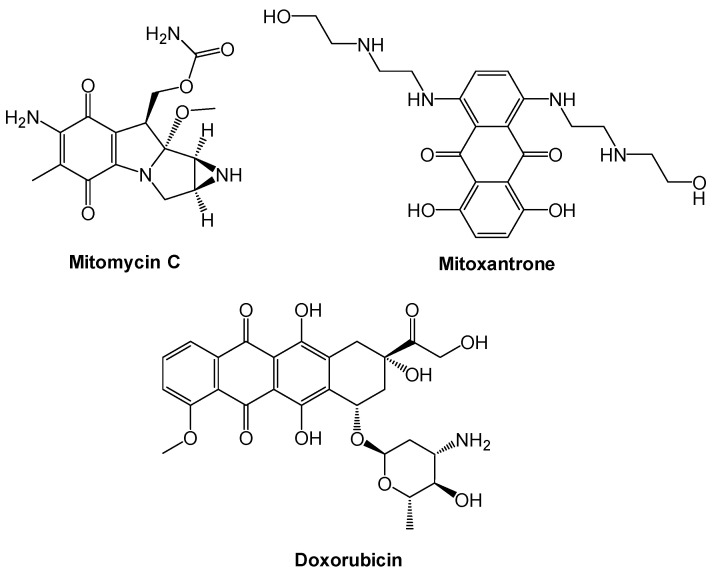
Mitomycin C, mitoxantrone, and doxorubicin, potential anticancer drugs the containing 1,4-quinone moiety.

**Figure 3:**
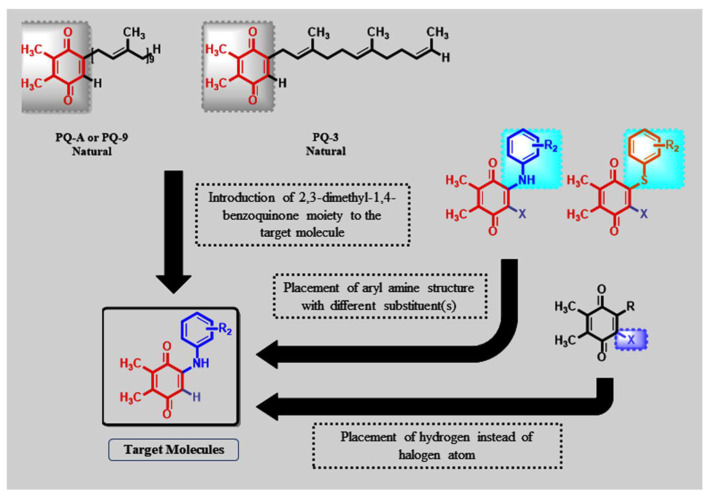
Design concept of **PQ** analogs based on our previous studies.

Regarding uses of important drugs for the treatment of cancer, mitomycin C, mitoxantrone, and doxorubicin draw attention and contain the 1,4-quinone moiety (Figure 2). After realizing the prominent anticancer activity of those structures, we created more using nature as a model, namely plastoquinones (**PQ**) discovered by Barr for electron transfer in photosynthesis in 1946 [24] containing both 2,3-dimethyl-1,4-benzoquinone structure and a side chain of nine isoprenyl group as PQ-A or PQ-9 [25] or shorter side chain such as PQ-3 with three isoprenyl side units, as shown in Figure 3 [26]. Studying common pharmacophoric features of **PQ** analogs has revealed that the presence of three main features, as shown in Figure 3, are important. These structures are (1) 2,3-dimethyl-1,4-benzoquinone as a main core structure, (2) aryl amines or thiols with different substituents, and (3) the presence of halogen (bromine and chlorine) or hydrogen atom since different combinations of these three main moieties affect the biological potential of molecules.

**Figure 4:**
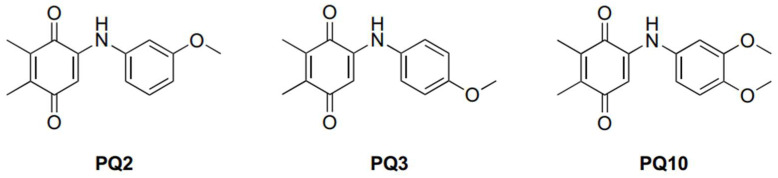
Structures of selected **PQ** analogs involved in the exploration.

In the period of 2017–2021, with the purpose of identification of new lead molecules based on natural products that specifically target leukemia cancer cell lines, we mainly focused on the quinones namely **PQ** analogs containing two important moieties (2,3-dimethyl-1,4-benzoquinone and primary/secondary amines or thiols) [27,28,29,30,31], encouraged by our previous results and experiences with 1,4-quinones [32,33,34]. Considering our previous findings that demonstrated greater anticancer activity by the introduction of an alkoxy group with a short alkyl chain [28,29], we also evaluated the effect of weak (methyl) or strong (methoxy) electron-donating group(s) to the **PQ** structure [27]. We recently reported the **PQ** analogs with aryl amines containing alkoxy substituent(s) as highly effective agents against two human cancer cell lines (K562 and Jurkat cells). Three **PQ** analogs (**PQ2**, **PQ3**, and **PQ10**) exhibited remarkable cytotoxic activities along with low toxicity towards the human peripheral blood mononuclear cell line (PBMC) (healthy), as shown in Table 1. Figure 4 illustrates the structures of selected **PQ** analogs. Moreover, the study showed that the selected **PQ** analog (**PQ2**) induced apoptosis in vitro in human chronic myelogenous leukemia cell lines exhibiting no toxicity in PBMC. In addition, the BCR-ABL1 mediated ERK pathway was clearly induced by **PQ2**. This analog (**PQ2**) enhanced the DNA cleaving capability with an iron (II) complex system [29].

In this study, three PQ analogs were evaluated in in vitro screening of anticancer activities against a panel of 60 cancer cell lines at 10 μM concentration. Based on these encouraging results, in the search for new lead molecules as antiproliferative agents, we focused on the discovery of the selected **PQ** analogs for their antiproliferative activity against the HCT-116 colorectal cancer cell line. Correspondingly, apoptotic effects, DNA binding potential and some essential absorption, distribution, metabolism and excretion (ADME) properties of the most effective anti-colorectal cancer agent were further investigated. 

## 2. Results 

### 2.1. Biological Activity

#### 2.1.1. In Vitro Screening of Antiproliferative Activity at One Dose

In order to study the full cytotoxic profile of three **PQ** analogs, the antiproliferative in vitro assay at a single dose of concentration (10 µM) was carried out by the National Cancer Institute (NCI) of Bethesda within the Developmental Therapeutics Program (DTP) against the panel of sixty human cancer cell lines, which includes nine tumor subpanels, namely: leukemia, non-small cell lung cancer (NSCLC), colorectal cancer, central nervous system (CNS) cancer, melanoma, ovarian, renal, prostate and breast cancer cell lines [35]. Herein, three **PQ** analogs namely **PQ2**, **PQ3**, and **PQ10** were selected by the NCI for in vitro disease oriented human cells screening panel assay. The cell growth/viability of **PQ2**, **PQ3**, and **PQ10** was screened using a sulphorhodamine B (SRB) test, thus both in vitro growth inhibition and lethality were determined based on the specified values, which were between 0 and 100 and less than 0, respectively.

**Figure 5:**
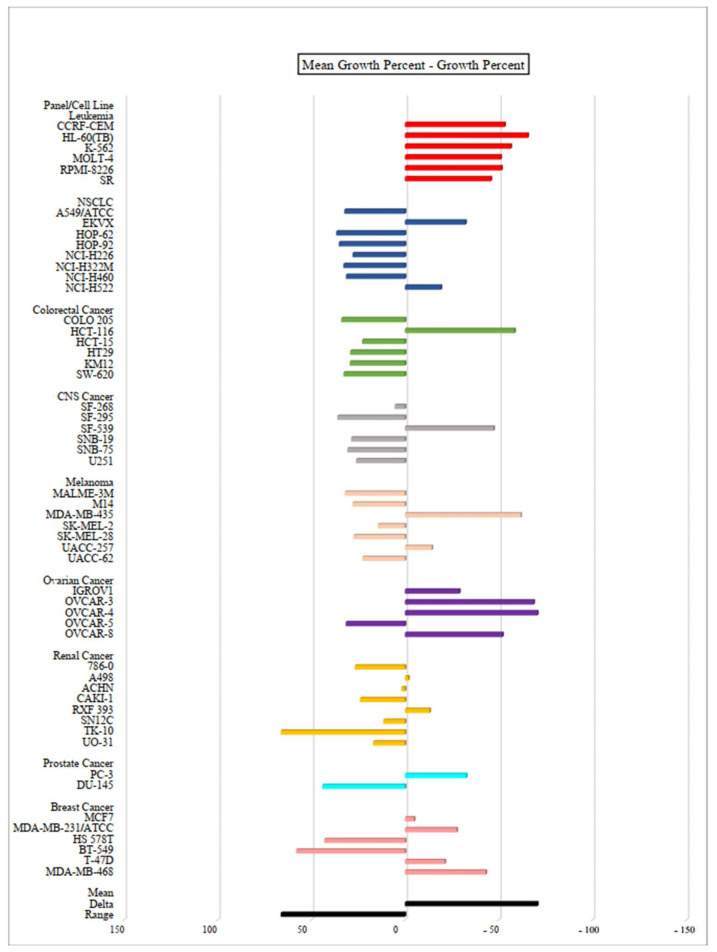
One-dose (10 µM) mean graph of **PQ2** against different cancer cell lines. All experiments were repeated one time.

**Figure 6:**
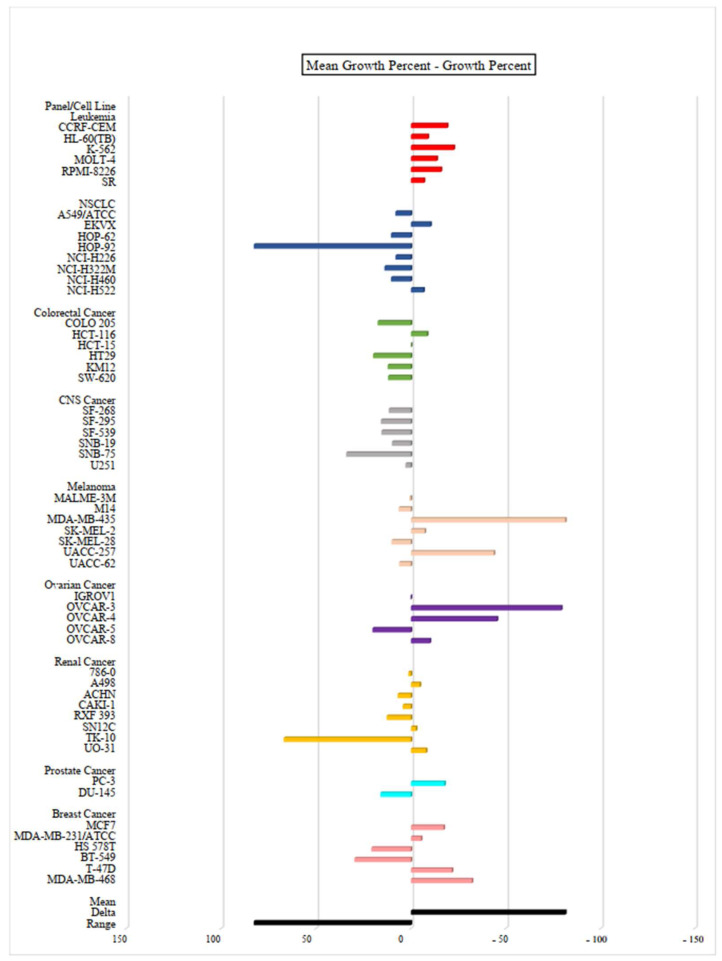
One-dose (10 µM) mean graph of **PQ3** against different cancer cell lines. All experiments were repeated one time.

**Figure 7:**
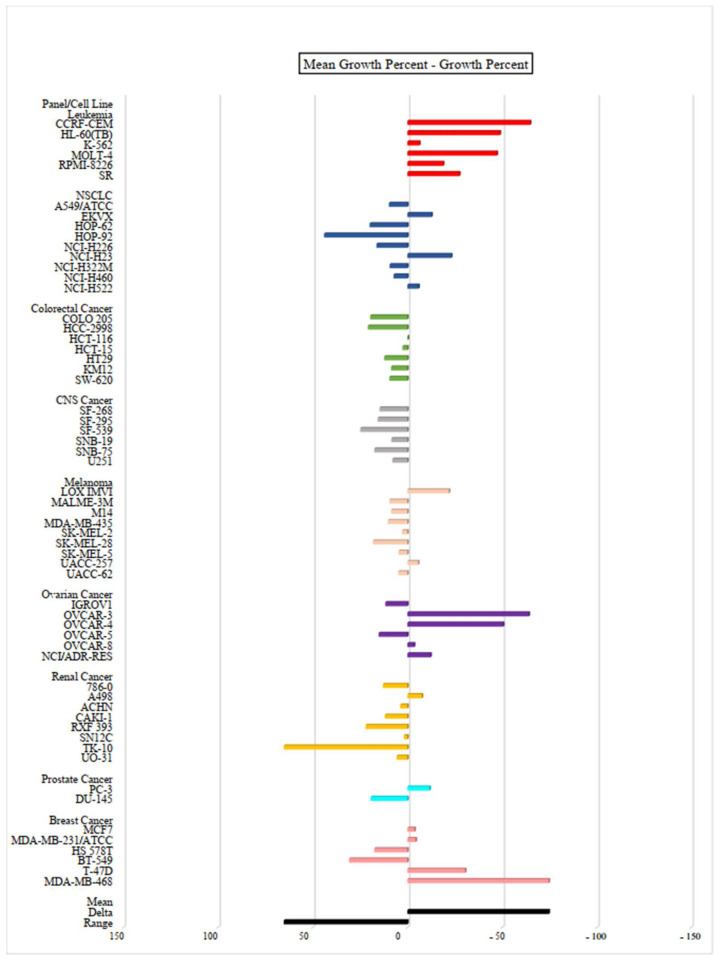
One-dose (10 µM) mean graph of **PQ10** against different cancer cell lines. All experiments were repeated one time.

The mean growth percent—growth percent of **PQ** analogs against for each cell line were depicted as bars in the one-dose mean graphs (Figure 5, Figure 6 and Figure 7 and Table 2). In general, **PQ3** and **PQ10** showed less inhibitory effects than **PQ2** on the tested cancer cell lines. **PQ3** presented the most significant inhibition against the MDA-MB-435 melanoma cell line with 92.20% inhibition percent followed by the OVCAR-3 ovarian cancer cell line (90.06%) (Table 2). Both analogs (**PQ2** and **PQ3**) showed good inhibitory profiles in MDA-MB-435 and OVCAR-3 cell lines. However, **PQ10** displayed the most significant inhibitory effects on MDA-MB-468 breast cancer cells with an inhibition percent of 81.85% followed by CCRF-CEM leukemia (72%) and OVCAR-3 ovarian cancer (71.28%) cell lines (Table 2). Among these analogs, **PQ2** was identified as the most effective antiproliferative agent with its inhibitory potency against different cancer cell lines with notable inhibition percent values in particular against HCT-116 colorectal cancer (89.46%), MDA-MB-435 melanoma (92.75%), OVCAR-3 ovarian cancer (99.71%) cell lines along with broad spectrum potent inhibitory effects on all the leukemia cell lines (Table 2). **PQ2** exhibited excellent to moderate antiproliferative activity to some leukemia cell lines supporting the previous report by our group regarding anti-leukemic effects of **PQ2** on K562 and Jurkat cells with IC_50_ values of 6.40 ± 1.73 μM and 7.72 ± 1.49 μM, respectively [30]. Previously, **PQ2** was also found to exert no cytotoxicity on healthy cell lines (PBMC) (>300 μM).

#### 2.1.2. Determination of Cell Viability

It was determined that antiproliferative effects of **PQ2** on HCT-116 cells were found to be more dominant than that of **PQ3** and **PQ10** compared to other cell lines in the panel of sixty human cancer cell lines. Due to significant inhibitory effects of **PQ2** against HCT-116 cells at one dose, its cytotoxic effects on HCT-116 cells were also evaluated by MTT (3-(4,5-dimethyl-2-thiazolyl)-2,5-diphenyltetrazolium bromide) assay at five concentrations (1, 3, 10, 30, and 100 μM) compared to cisplatin. Cisplatin was chosen as a standard agent due to frequent use in the treatment of colorectal cancer in spite of rapid development of resistance [36].

**Figure 8:**
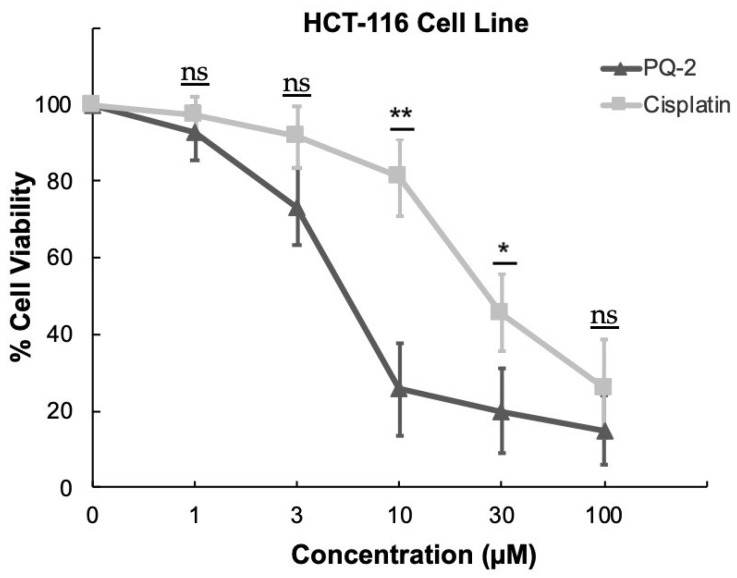
The anticancer effects of **PQ2** and cisplatin at varying concentrations on HCT-116 cells. Data are representative of the mean of three separate experiments and are reported at the ±SD and *p* values were determined using Student’s *t* test (* *p* < 0.05, ** *p* < 0.01, ns: not statistically significant).

According to the results, **PQ2** exhibited significant cytotoxic effect against HCT-116 cells with an IC_50_ value of 4.97 ± 1.93 μM compared to control drug cisplatin (IC_50_ = 26.65 ± 7.85 μM) (*p* < 0.05). Moreover, significant cytotoxic effects of **PQ2** on HCT-116 cells at varying concentrations compared to cisplatin are outlined in Figure 8. The apparent decline in percentage of viable cells was observed between 3 and 10 μM with **PQ2** treatment, whereas a similar decline appeared between 10 and 30 μM with cisplatin treatment. 

#### 2.1.3. Determination of Cell Death

**Figure 9:**
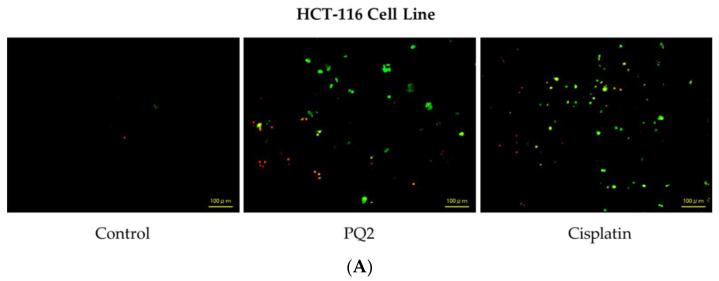

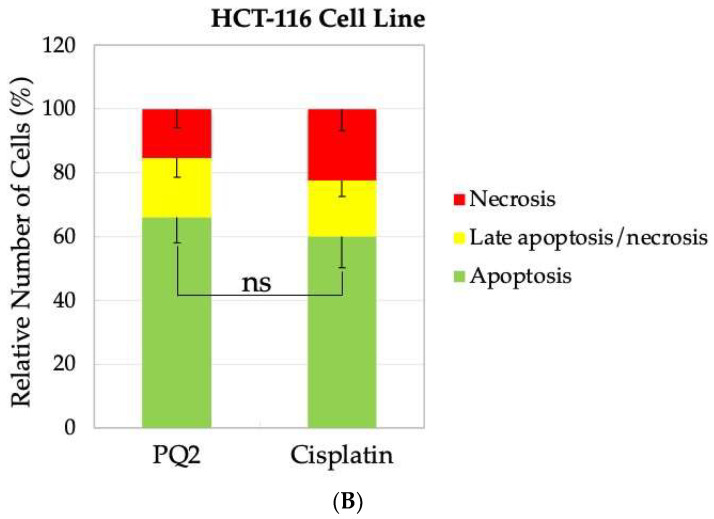
Alteration of HCT-116 cells following exposure to the IC_50_ concentration of the control (DMSO), **PQ2**, and cisplatin (**A**) for 12 h. The percentage of apoptotic (green), late apoptotic or necrotic (yellow) and necrotic (red) cells (**B**) was determined by analyzing 100 randomly chosen stained cells in each experiment. Data from three independent experiments are shown as means ± SD, and *p* values were determined using Student’s t test (ns: not statistically significant).

In the current study, the significant anticancer potential of **PQ2** on the HCT-116 cells encouraged us to investigate its apoptotic and necrotic activity in HCT-116 cell line using the annexin V/ethidium homodimer III staining method. This method indicates that apoptotic, necrotic or late apoptotic, and necrotic cells stain positive for green, yellow and red, respectively. These alterations in cells are observed with a fluorescence microscope. It was determined that HCT-116 cells-treated with **PQ2** and cisplatin underwent apoptosis mainly in less time (Figure 9A). According to the results, **PQ2** revealed 66% apoptotic, 19% late apoptotic/necrotic and 15% necrotic effects when compared with cisplatin (60%, 18% and 22%, respectively) at 12 h (Figure 9B). This outcome pointed out that **PQ2** significantly enhanced apoptosis in HCT-116 cells.

**Figure 10:**
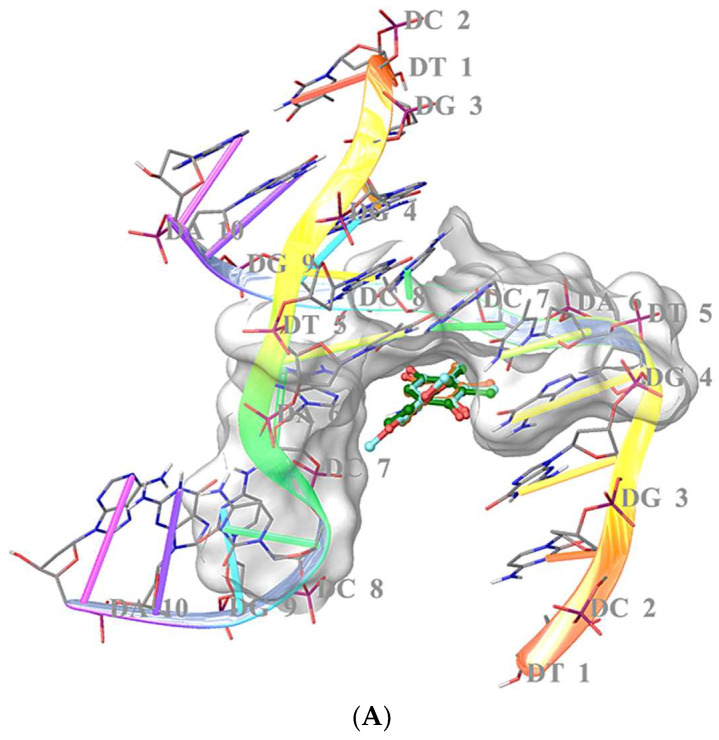

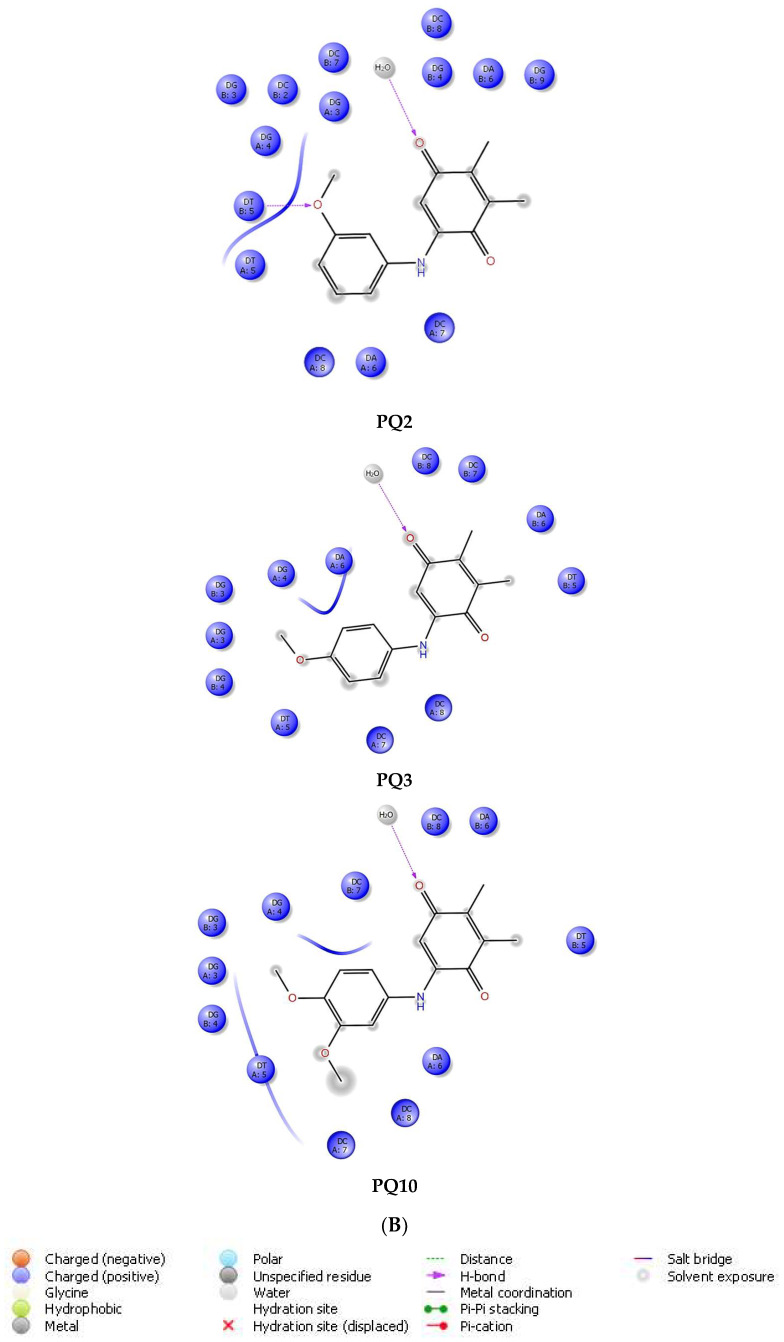
Docking poses (**A**) and interactions (**B**) of **PQ2**, **PQ3** and **PQ10** (ligands are highlighted in dark green-, orange- and turquoise-colored sticks) in the minor groove of the double helix of DNA (PDB ID: 2GWA).

It is well-known that there is a correlation between DNA cleavage and apoptosis. It was previously shown that **PQ2** was able to cleave DNA in the presence of FeSO_4_, H_2_O_2_ and ascorbic acid [29]. Due to significant DNA cleaving activity of **PQ2**, molecular docking studies were carried out to understand the binding capacity of **PQ2** to DNA (PDB ID: 2GWA) in comparison with **PQ3** and **PQ10** [37]. Results showed that **PQ2** displayed higher binding affinity to DNA, forming a hydrogen bond with DT-5 through its methoxy moiety in the minor groove of the double helix of DNA, whereas **PQ3** and **PQ10** presented no interactions (Figure 10A,B). The docking scores of the compounds were detected to range from −5.628 to −5.848 kcal/mol (Table 3). 

### 2.2. In Silico Prediction of Pharmacokinetic Properties

Some risks and physicochemical properties of **PQ2** were estimated by ADMET Predictor 9.0 and compatibility to the Lipinski rule of 5 was confirmed in our previous study [29]. In recent work, some other essential ADME parameters of **PQ2** such as predicted octanol/water partition coefficient (QPlogPo/w), predicted aqueous solubility (QPlogS), human serum albumin binding (QPlogKhsa), predicted brain/blood partition coefficient (QPlogBB) were in silico ascertained.

The QPlogPo/w and the QPlogS values of **PQ2** (2.129 and −3.602, respectively) were detected within the range (−2 to 6.5 and −6.5 to 0.5, respectively). **PQ2** was also found to reveal significant human serum albumin binding with a QPlogKhsa value of −0.133 within the specified range (−1.5 to 1.5). The QPlogBB value of **PQ2** (−0.465) was detected in an optimal range of the specified values (−3 to 1.2) indicating its ability to cross the blood–brain barrier (BBB). Furthermore, **PQ2** presented remarkable human oral absorption (95.119%) on a 0–100% scale (>80% is high; <25% is poor).

## 3. Discussion

Gastrointestinal cancers are globally responsible for one in three cancer deaths. Within the types of gastrointestinal cancers, colorectal cancer is the third most common type in cases and the second most common type in deaths. In the statistics published by the International Agency for Research on Cancer (IARC) in 2020, about 1.0 million new cases (10.6) in males and 0.8 million new cases in females in all ages were diagnosed with this cancer in the worldwide. Thus, colorectal cancer was slightly more common in males than in females [38].

**Figure 11:**
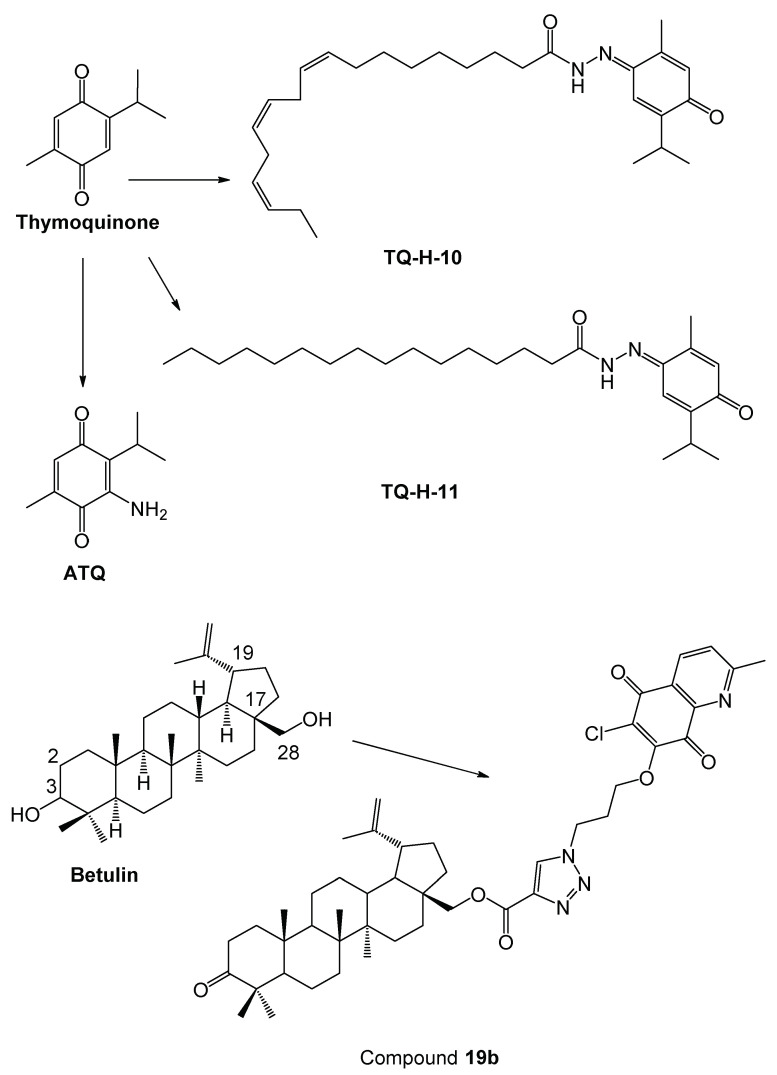
Thymoquinone and betulin analogs as anti-colorectal cancer agents.

In previous studies related to the anti-colorectal effects of natural quinone derivatives, significant results were obtained. However, in general the most potent anticancer agents were found to be cytotoxic against healthy cells. Thymoquinone (Figure 11) was searched for its anticancer effects on HT-29 human colorectal adenocarcinoma cells and revealed anticancer activity based on the trypan blue exclusion method [39]. Thymoquinone derivatives were also investigated for their anti-colorectal cancer properties in distinct studies. Thymoquinone-4α-linolenoylhydrazone (**TQ-H-10**) (Figure 11) and thymoquinone-4-palmitoylhydrazone (**TQ-H-11**) (Figure 11) derivatives were found to be effective towards HCT-116 cells. [40]. In another study [41], 3-aminothymoquinone (**ATQ**) (Figure 11) was synthesized from starting compound thymoquinone and searched for antiproliferative activity against an SW620 colorectal adenocarcinoma cell line with a WI38 healthy fibroblast cell line. **ATQ** displayed cytotoxic effects against colorectal cancer cells, whereas this compound also showed cytotoxic affects against WI38 cells. A natural active compound, betulin (Figure 11), is obtained from the bark of white birch trees. 1,4-Quinone-based betulin derivatives were detected to be effective towards different cancer cells including colorectal cancer cells. 1,4-Quinone-based betulin derivatives were synthesized and evaluated for their anticancer effects against seven human cancer cell lines including Caco-2 colorectal cancer cells and HFF1 normal fibroblast cells. 28-(3-(6-chloro-2-methyl-5,8-quinolinedione-7-yloxy)-propyl-1*H*-1,2,3-triazol-4-ynoiloxy)-3-oxolup-20(29)-en (**19b**) (Figure 11) exhibited the most potent anti-colorectal activity compared to cisplatin, whereas **19b** also showed cytotoxic effects against HFF1 cells [42]. Recent study showed that **PQ2** displayed notable inhibition against HCT-116 cell line though **PQ3** and **PQ10** showed no cytotoxicity, indicating more selective anticancer action of **PQ2** towards HCT-116 cells compared to **PQ3** and **PQ10**. This outcome pointed out that the addition of the methoxy substitution to the anilino group led to a decrease in the anticancer activity, as shown in **PQ10** compared to **PQ2** and **PQ3**, whereas one methoxy substitution at the meta position on the anilino ring (**PQ2**) increased anti-colorectal cancer activity when compared with one methoxy substitution at the para position (**PQ3**). Furthermore, when compared with aforementioned studies, **PQ2** revealed superior cytotoxicity against colorectal cancer cells and selective anti-colorectal cancer activity exerting no cytotoxicity to healthy cells.

Apoptosis is pivotal in tissue homeostasis, particularly in gastrointestinal tract and immune system. In addition, apoptosis induction in cancer cells is a promising strategy for the treatment of cancer. In the early stages of cancer, cancer cells are more sensitive to agents, which stimulate apoptosis. Then, these cells start to become resistant to apoptotic stimuli [43,44,45,46]. Therefore, there is an urgent need to discover more potent anticancer agents that also induce apoptosis in cancer cells. Some quinone-based natural compounds were evaluated for their apoptotic effects on colorectal cancer cells. Seetha et al. [21] performed morphological analysis, cell cycle regulation, and dual staining using acridine orange and ethidium bromide in control and treated cells revealing the apoptotic potential of juglone (Figure 1) along with indomethacin, which was found to be effective in the proliferation of colorectal cancer cells. The same research group indicated that juglone diminished the inflammatory activity and stimulated apoptosis together with indomethacin [22]. Previously, we also reported that **PQ2** demonstrated 69% apoptotic, 20% late apoptotic/necrotic, and 11% necrotic effects on K562 cells at 6 h [29]. This current study pointed out that **PQ2** also significantly enhanced apoptosis in colorectal cells when compared with cisplatin.

Molecular docking studies were performed for **PQ2** to explore its binding potential in the minor groove of the double helix of DNA. In our previous studies, we also performed molecular docking studies for quinone-based compounds with the most significant DNA cleaving abilities. 7-Chloro-6-(3,5-dimethylphenyl)amino-5,8-quinolinequinone (**AQQ15**) [47] and 6-(benzo[*d*][1,3]dioxol-5-ylamino)-7-chloro-5,8-quinolinequinone (**AQQ13**) [48] formed important interactions with DNA. In the current work, **PQ2** also formed a key hydrogen bonding through its methoxy moiety in the minor groove of the double helix of DNA. On the contrary, **PQ3** and **PQ10** formed no interactions. In general, the docking scores of the compounds were consistent with the biological data. The highest docking score, belonging to **PQ2** explained its high binding capacity to DNA. These outcomes indicated that the strong effect of **PQ2** against HCT-116 cell line would be related to strong DNA binding of **PQ2**. Moreover, it can be concluded that the presence of a methoxy substituent at third position of anilino ring played a fundamental role in binding with DNA when compared with 4-methoxyanilino and 3,4-dimethoxyanilino substituted compounds (**PQ3** and **PQ10**, respectively).

The logPo/w is a crucial pharmacokinetic determinant, which affects membrane permeability, metabolism, binding potency to the target, bioavailability and toxicity of compounds [49]. In order to show its physiological effect, compounds should also transport to the site of action in particular in aqueous environments as the human body consists of approximately 60% water. Furthermore, compounds with low aqueous solubility are prone to demonstrate poor uptake, lack of pharmacological effect, manufacturing and storage problems [50]. Moreover, the determination of QPlogKhsa acts a crucial role for a suitable pharmacokinetic profile of compounds at an acceptable dose and dose frequency influencing volume of distribution and half-life of them [51]. The BBB restrains drug entry from blood into brain by multiple mechanisms [52]. The QPlogPo/w, the QPlogS, the QPlogKhsa and the QPlogBB values of **PQ2** were detected in an optimal range of the specified values with a high human oral absorption percentage indicating that **PQ2** possessed orally bioavailable drug-like properties.

## 4. Materials and Methods

### 4.1. Chemistry

The synthetic protocol and spectral data of **PQ2**, **PQ3**, and **PQ10** were reported previously [29].

### 4.2. Biological Evaluation

#### 4.2.1. In Vitro Antiproliferative Activity at Single-Dose Concentration by NCI

**PQ** analogs were submitted to NCI, Bethesda, USA and, as per standard protocol of NCI, all compounds were evaluated for their antiproliferative activity at single dose assay (10 µM concentration in DMSO) on a panel of sixty cancer cell lines derived from leukemia, lung, colorectal, CNS, melanoma, ovarian, renal, prostate, and breast cancers as per protocol [43]. Tested compounds were added to the microtiter culture plates followed by incubation for 48 h at 37 °C. SRB, a protein binding dye, was used for end point determination. The percent of growth of the treated cells was determined in comparison to the untreated control cells and the results of each tested compound were reported. Data from one-dose experiments pertain to the percentage growth at 10 μM [53,54].

#### 4.2.2. Cell Culture and Drug Treatment

The HCT-116 cell line (provided by the RIKEN BRC through the National Bio-Resource Project of the MEXT/AMED, Japan (RCB2979)) was incubated in Dulbecco’s modified Eagle’s medium (DMEM) (Wako Pure Chemical Industries, Osaka, Japan). DMEM was supplemented with 10% fetal bovine serum (FBS) (Sigma Aldrich, St. Louis, MO, USA) and 89 μg/mL streptomycin (Meiji Seika Pharma, Tokyo, Japan) at 37 °C in humidified 5% CO_2_ atmosphere. HCT-116 cells were cultured in 24-well plate (Iwaki brand Asahi Glass Co., Chiba, Japan) at 4 × 10^4^ cells/mL concentration for 48 h (the optimum cell number was verified associated with previous study) [55]. The stock solution of **PQ2** and cisplatin in concentrations ranging from 0.1 to 10 mM were prepared in DMSO (Wako Pure Chemical Industries, Osaka, Japan) and further diluted with fresh culture medium. The concentration of DMSO in the final culture medium was 1% which had no effect on the cell viability.

#### 4.2.3. MTT Assay

MTT (Dojindo Molecular Technologies, Kumamoto, Japan) was used to determine the effects of **PQ2** and cisplatin on cell viability as previously described [56,57]. HCT-116 cells were treated with **PQ2** and cisplatin at five concentrations (1, 3, 10, 30, and 100 μM) for 48 h at 37 °C and then stained with MTT solution and further incubated for 4 h. Afterwards, supernatants were removed and 100 μL DMSO was added to each well. The absorbance of the solution was analyzed using a plate reader Infinite M1000 (Tecan, Mannedorf, Switzerland). All experiments were carried out as three times and IC_50_ values were determined as the drug concentrations that decreased absorbance to 50% of control values.

#### 4.2.4. Detection of Cell Death

The HCT-116 cell line was incubated with **PQ2** and cisplatin at IC_50_ concentration for 12 h before the apoptotic/necrotic/detection kit (PromoKine, Heidelberg, Germany) was applied with some modifications to the manufacturer’s directions [55,58]. Then, HCT-116 cells, which were treated with appropriate content including binding buffer and staining solution, were detected by an all-in-one fluorescence microscope Biorevo Fluorescence BZ-9000 (Keyence, Osaka, Japan). Based on our previous studies [59], the number of apoptotic, late apoptotic or necrotic and necrotic cells was counted relevant to the staining with annexin V and ethidium homodimer III.

### 4.3. Molecular Docking Studies

**PQ2** was sketched and cleaned in the Maestro molecular modeling workspace followed by energy minimization in the ligand preparation program of Schrödinger’s Maestro molecular modeling package (Schrödinger Release 2016-2: Schrödinger, LLC, New York, NY, USA) using an Optimized Potential Liquid Simulations (OPLS_2005) force field at physiological pH (pH = 7.4). The X-ray crystallographic structure of DNA was acquired from the PDB server (PDB ID: 2GWA) [37] and optimized for docking analysis in protein preparation module of Schrödinger software. In molecular docking simulations, Grid Generation and Glide/ XP docking protocols were implemented [47,48].

### 4.4. In Silico ADME Estimation

Some pharmacokinetic characteristics of **PQ2** were calculated by the QikProp module of Schrödinger software (Schrödinger Release 2016-2: QikProp, Schrödinger, LLC, New York, NY, USA, 2016) [47,48].

## 5. Conclusions

The potential cytotoxic effects of a series of **PQ** analogs bearing alkoxy substituted anilino moiety were previously heralded by our research team. Among these analogs, **PQ2**, **PQ3** and **PQ10** were chosen by NCI for in vitro screening of antiproliferative activity against a wide range of cancer cell lines stemming from nine distinct cancer types. The 3-Methoxyphenyl carrying compound (**PQ2**) exerted superior antiproliferative activity against the HCT-116 cell line compared to **PQ3** and **PQ10**. The results of MTT assay also supported the promising anti-colorectal cancer effects of **PQ2** at different concentrations. Further studies also indicated that **PQ2** induced apoptosis in colorectal cancer cells compared to cisplatin and it was found to establish proper hydrogen bonding with DNA reinforcing the DNA cleavage-associated cell death. Some in silico predicted ADME parameters reflected that **PQ2** was endowed with orally bioavailable drug-like properties. All these outcomes shed light on future mechanistic studies for **PQ2** owing to its notable anticancer activity against colorectal cancer. To the best of our knowledge, one of the more significant findings to emerge from this study is that this is the first study showing the anti-colorectal cancer activity of **PQ** analogs. This finding has important implications for developing new agents based on **PQ** for colorectal cancer treatment instead of the known inhibitors.

## Figures and Tables

**Table 1:** The cytotoxic effects of **PQ2**, **PQ3**, and **PQ10** on K562, Jurkat, MT-2 and PBMC cells compared to imatinib.

ID	Substitution Groups				Cell Type (IC_50_, μM)			
	R_1_	R_2_	R_3_	R_4_	K562 ^a^	Jurkat ^a^	MT-2 ^a^	PBMC ^a^
**PQ2**	H	OCH_3_	H	H	6.40 ± 1.73	7.72 ± 1.49	>100	>300
**PQ3**	H	H	OCH_3_	H	9.66 ± 2.31	22.75 ± 1.93	53.96 ± 3.81	72.68 ± 6.51
**PQ10**	H	OCH_3_	OCH_3_	H	8.91 ± 1.26	14.47 ± 1.35	35.79 ± 0.89	69.35 ± 7.12
Imatinib ^b^					7.47 ± 2.22	9.49 ± 2.46	22.09 ± 1.76	39.81 ± 4.38

^a^ Cell lines include chronic myelogenous leukemia (K562), other leukemias (Jurkat and MT-2), and peripheral blood mononuclear cells (PBMC). ^b^ Used as a reference.

**Table 2:** Antiproliferative activity data as per single-dose assay at 10 µM concentration as cell growth percent of **PQ** analogs. All experiments were repeated one time.

Panel/Cancer Cell Line	Compounds		
	PQ2	PQ3	PQ10
	Growth Percent		
**Leukemia**			
CCRF-CEM	15.84	70.07	28.00
HL-60(TB)	3.49	80.23	43.99
K-562	12.54	66.58	86.47
MOLT-4	17.89	75.57	45.57
RPMI-8226	17.52	73.34	73.88
SR	23.16	82.29	65.36
**NSCLC**			
A549/ATCC	101.36	97.30	102.53
EKVX	36.72	78.84	80.02
HOP-62	105.69	99.66	112.71
HOP-92	104.31	171.92	136.73
NCI-H226	97.00	97.24	109.09
NCI-H23	ND *	ND *	69.62
NCI-H322M	101.88	103.08	102.13
NCI-H460	100.51	99.63	100.07
NCI-H522	49.81	82.47	86.90
**Colorectal Cancer**			
COLO 205	103.02	106.66	112.41
HCC-2998	ND *	ND *	113.67
HCT-116	10.54	80.60	92.46
HCT-15	91.87	89.04	95.39
HT29	98.29	109.10	105.03
KM12	98.44	101.37	101.36
SW-620	101.81	101.18	102.32
**CNS Cancer**			
SF-268	74.53	100.81	107.49
SF-295	105.11	104.94	108.44
SF-539	21.69	104.69	117.66
SNB-19	97.72	99.17	101.23
SNB-75	99.78	123.25	110.24
U251	95.10	92.04	100.71
**Melanoma**			
LOX IMVI	ND *	ND *	70.80
MALME-3M	101.18	89.92	102.29
M14	97.05	95.56	101.40
MDA-MB-435	7.25	7.80	103.13
SK-MEL-2	83.57	81.83	95.65
SK-MEL-28	96.53	99.39	110.98
SK-MEL-5	ND *	ND *	97.50
UACC-257	54.67	45.42	87.07
UACC-62	91.77	95.37	97.60
**Ovarian Cancer**			
IGROV1	39.99	89.25	104.41
OVCAR-3	0.29	9.94	28.72
OVCAR-4	−1.68	43.81	42.19
OVCAR-5	100.60	109.36	107.95
OVCAR-8	16.95	79.12	89.21
NCI/ADR-RES	ND *	ND *	80.54
SK-OV-3	ND *	ND *	ND *
**Renal Cancer**			
786-0	95.76	90.51	105.71
A498	67.18	84.44	85.07
ACHN	70.90	96.11	96.60
CAKI-1	93.02	93.46	104.67
RXF 393	55.80	101.91	114.84
SN12C	80.52	86.43	94.65
TK-10	135.29	156.13	158.01
UO-31	86.04	81.15	98.44
**Prostate Cancer**			
PC-3	36.32	71.45	81.05
DU-145	113.12	105.22	112.19
**Breast Cancer**			
MCF7	64.11	71.87	88.94
MDA-MB-231/ATCC	41.39	83.80	88.36
HS 578T	112.09	109.96	110.28
BT-549	127.05	118.89	123.57
T-47D	47.75	67.51	62.22
MDA-MB-468	25.85	56.98	18.15

* “ND” means not determined.

**Table 3:** Docking score (kcal/mol), glide gscore (kcal/mol) and glide emodel (kcal/mol) results of **PQ2**, **PQ3**, and **PQ10** for DNA (PDB ID: 2GWA).

Compound	2GWA		
	Docking Score	Glide Score	Glide Emodel
**PQ2**	−5.848	−5.848	−48.650
**PQ3**	−5.628	−5.628	−43.682
**PQ10**	−5.833	−5.833	−44.605

## Data Availability

Not applicable.

## Sample Availability

Samples of the compounds **PQ2**, **PQ3**, and **PQ10** are available from the authors.
